# School Absence and Mental Health in a Help-Seeking Sample From Child and Adolescent Psychiatry

**DOI:** 10.1177/13591045251400395

**Published:** 2025-11-24

**Authors:** Bas T. H. de Veen, Martin Knollmann, Martine van Dongen-Boomsma, Pierre C. M. Herpers, Ingo Spitczok von Brisinski, Johannes Hebebrand, Ron H. J. Scholte, Wouter G. Staal, Volker Reissner

**Affiliations:** 1 Behavioural Science Institute, Radboud University, Nijmegen, The Netherlands; 2 Child and Adolescent Psychiatry, Karakter, Nijmegen, The Netherlands; 3 Department of Child and Adolescent Psychiatry, Psychosomatics & Psychotherapy, University of Duisburg-Essen, Essen, Germany; 4 University Hospital Essen, Essen, Germany; 5 Depression Expertise Centre for Youth, GGZ Oost Brabant, Oss, The Netherlands; 6 Child and Adolescent Psychiatry, LVR-Clinic, Viersen, Germany; 7 Department of Psychiatry, Radboud University Medical Centre, Nijmegen, The Netherlands; 8 Leiden Institution for Brain and Cognition, Leiden, The Netherlands

**Keywords:** school absence, problematic school absence, international, child and adolescent psychiatry, mental health, clinical psychiatric population

## Abstract

**Objective:**

Problematic school absence (PSA) can severely impact children’s mental health. Research on the prevalence of PSA in child and adolescent psychiatry is still limited. This study explores the prevalence and severity of PSA in Dutch and German young people with mental health problems.

**Methods:**

This study included 354 patients aged 6-20 years (*M* = 13.0; *SD* = 3.5) from three psychiatric clinics in the Netherlands and Germany (151 Dutch and 203 German children), recruited between March and June 2019. Parents completed the Strengths and Difficulties Questionnaire and the School-Non-Attendance-ChecKlist. The study analysed PSA and associated factors.

**Results:**

School absence occurred in 66.9% of the sample. Any PSA was significantly associated with inpatient treatment, conduct problems and comorbidity, while attention-deficit/hyperactivity disorder, and other behavioural disorders were negatively associated. Severe PSA (missing 8 or more of 20 school days) was associated with higher age, inpatient treatment, and phobic anxiety disorders.

**Conclusion:**

This study showed a high prevalence of PSA among children with mental health problems in an international sample. Given its impact on children’s development and future prospects, addressing PSA should be a priority for mental health professionals working with young people with mental health problems.

## Plain Language Summary

*Why was the study done?* Missing school can affect children’s mental health, learning, and future. Children with mental health problems are more likely to miss school, but research on how often and why this happens is limited. This study explored how often young people in mental health care are absent from school and what factors might be linked to that. *What did the researchers do?* The researchers studied 354 children and teenagers aged 6 to 20 who were receiving mental health care in the Netherlands and Germany. Some stayed in a clinic, while others lived at home. Parents filled in forms about how many school days their child missed and the reasons why. The researchers also looked at the mental health diagnoses of each child to see if there were patterns linked to school absence. *What did the researchers find?* Two out of three children missed school at least once in the past four weeks. One in four missed 8 or more days, and one in ten missed every day. The main reasons were school refusal, meaning, not wanting or being too anxious to go, and being sent home by the school. Older children, those in clinics, and those with multiple mental diagnoses were more likely to miss school. Children with conduct or anxiety disorders were also more at risk. However, children with attention-deficit and hyperactivity problems missed less school, possibly because many of them were receiving treatment like medication. *What do the findings mean?* School absence is very common among children and teenagers receiving mental health care, especially in older children, those with multiple mental health problems, and those in clinics. Mental health professionals and schools should work together to support these children. Helping them attend school regularly can improve their well-being and future opportunities. These findings can also help guide future research and care to reduce the risk of long-term school problems.

## Introduction

Worldwide, school absence has a negative impact on children’s mental health (Kearney, 2008a; Klein et al., 2020; Knollmann et al., 2010) and their development, impacting both short- and long-term prospects (Gottfried, 2010; Schoeneberger, 2012). Regular school attendance provides daily structure, supports learning, and fosters peer relationships, thereby contributing to children’s cognition, socialization and emotional development (Sylva, 1994). Prolonged school absence disrupts these developmental processes (Garland, 2001), and many children who are frequently absent from school also have more mental health problems (Egger et al., 2003; Heyne & Sauter, 2013). Hence, mental health problems themselves can both influence school attendance and be affected by it. Although not all mental health problems lead to school absence, various interconnected and reinforcing factors can contribute to it. Understanding how school absence manifests in children with mental health problems is essential for gaining insight into this complex issue.

School absence can be classified into two main types (Heyne et al., 2019a): (a) non-problematic school absence (NPSA) and (b) problematic school absence (PSA) (Heyne et al., 2019a). NPSA refers to permitted or authorized absence, such as missing school due to physician appointments, absence based on a sick-note, special/religious holidays, officially closed school, excusable family situations, or weather conditions. PSA denotes unauthorized absence which is further subdivided in four types of PSA: (a) *School refusal* refers to the child being anxious or upset about school and reluctant or refused to go to school, (b) *truancy* constitutes the child skipping class or leaving school without permission, (c) *school withdrawal* refers to parents giving the child a day off/extra holidays or keeping them at home, and (d) *school exclusion* is defined as a formal removal of a student from the school environment due to behavioural issues. Although NPSA and PSA are differentiated by permission, any form of school absence can affect children’s mental health and development (Gallé-Tessonneau et al., 2019; Klein et al., 2022).

In the general population, school refusal affects 1-7% of youths (Egger et al., 2003; Havik et al., 2015; Heyne & King, 2004), and chronic school absence (i.e., an absence of more than 10% of school days (Dutch Support Centre for Inclusive Education, 2024; UK Department for Education, 2019; US Department of Education, 2016)) involves 1 in 15 students (Dutch Support Centre for Inclusive Education, 2024; UK Department for Education, 2019; US Department of Education, 2016). As classifications include difficulties across multiple settings (i.e., home, school and peer interactions) (American Psychiatric Association, 2013; World Health Organization, 1992), higher school absence rates are expected, with school refusal in clinical psychiatric populations estimated to range from 5-16% among children and adolescents (Al Husni Al Keilani & Delvenne, 2021; Burke & Silverman, 1987; Honjo et al., 1992; McShane et al., 2001; Roué et al., 2021). Furthermore, as different mental health problems present unique challenges, school absence types may vary depending on the social difficulties.

**Figure 1.**
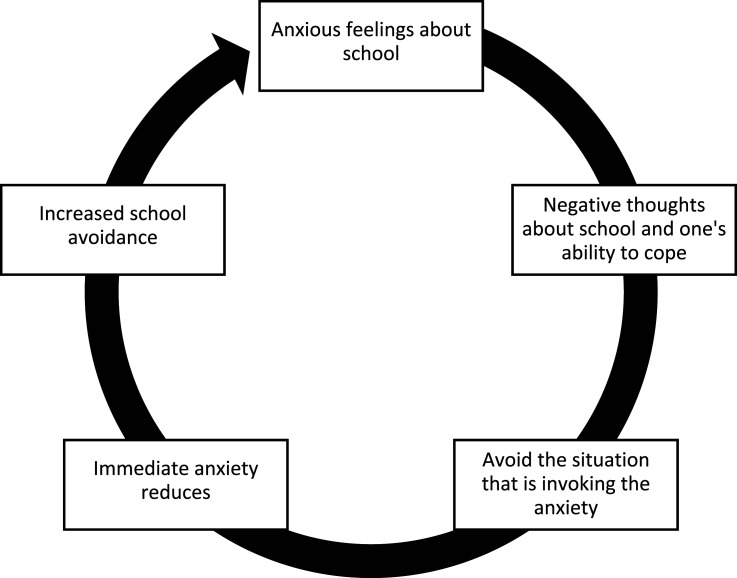
Illustrative Cycle Showing How School Absence can Develop Into a Self-Perpetuating Pattern (Adapted From West Sussex County Council (2022))

In particular, children with mental health problems such as neurodevelopmental-, emotional-, or disruptive behavioural disorders are significantly more likely to miss school (Finning et al., 2020; Lawrence et al., 2019; Totsika et al., 2020). Notably, students with mental health problems, with attention-deficit/hyperactivity disorder and anxiety as most common, were found to be absent twice as often as their peers without mental health problems (Lawrence et al., 2019). Similarly, emotional problems such as anxiety and depression are strongly associated with school absence, with individuals with depression being 3.4 times more likely to miss school (Finning et al., 2020). Furthermore, children with autism spectrum disorders missed an average of 5 days out of 23 possible school days, finding that parent-reported absences showed 43% were due to school refusal, 9% each contributed to school exclusion and school withdrawal, with truancy nearly absent. Additionally, 32% of absences were due to non-problematic reasons, mainly medical appointments (Totsika et al., 2020). Given the strong connection and negative transactional cycle (Adams, 2022; John et al., 2022; Lawrence et al., 2019) between PSA and mental health problems (see example in Figure 1), it is essential to address these issues in mental health care to better understand how social difficulties manifest in different types of school absence.

While most research has focused on the general population (Finning et al., 2019; Johnsen et al., 2022), studies on PSA within clinical psychiatric populations remain limited. This study aims to explore PSA types in clinical outpatient and inpatient psychiatric populations, assessing its prevalence, duration and associations with various mental health classifications and related factors. These insights aim to enhance clinicians’ awareness of school absence among children and adolescents receiving mental health care.

## Methods

### Procedure

This study used an exploratory retrospective cross-sectional study design investigating PSA among Dutch and German children receiving clinical child and adolescent psychiatric care. Data were collected at a single time point to assess associations between school attendance and psychometric and demographic variables.

Participants were referred to child and adolescent psychiatric clinics by medical doctors and youth care institutions. Participants were recruited from Dutch and German clinics in the border region between March and June 2019. A convenience sampling method was used for this study, randomly selecting participants who were readily accessible and willing to participate. In the Dutch sample, all newly registered patients received a digital invitation to participate. Questionnaires were administered via computer assisted web interviews. In the German sample, parents from newly registered patients were randomly approached in person by clinicians and invited to participate in the study.

Parents provided informed consent as they completed the parent-rating forms, and the study was approved by the Dutch and German Institutional Review Boards associated with the participating child and adolescent psychiatric clinics.

### Measures

#### Demographic Characteristics

Information on age, sex, educational level, school grade, age at school entry, and number of years repeated was obtained from parent reports. The International Standard Classification of Education (ISCED) was used as a standardized framework for categorizing and comparing educational levels across countries (UNESCO, 2012).

#### School Absence

The School Non-Attendance ChecKlist (SNACK; Heyne et al. (2019b)) is a scale that categorizes school absence. Duration ratings were requested for both whole and half days during the 20 school days prior to the assessment. Parents indicate the reasons for each missed day by selecting one of 15 predefined options (see also Table 1). Reasons for school absence are classified into five possible categories, which are broadly grouped into two overarching types: NPSA and PSA. NPSA (SNACK reasons 1, 2, 8, 9, 10, 11 and 14) refers to absence authorised by school (Heyne et al., 2019b). PSA includes school refusal (SNACK reason 3), truancy (SNACK reason 4), school withdrawal (SNACK reasons 5, 6,7), and school exclusion (SNACK reasons 12, 13) (Heyne et al., 2017, 2019b; Kearney & Silverman, 1993).

**Table 1.** School Non-Attendance ChecKlist

SNACK reasons	Examples
1. Child had an appointment	A doctor’s appointment or an appointment with a specialist
2. Child was sick	Had a cold or flu; had asthma; was in hospital
3. Child was reluctant or refused	Said it was hard to go to school or to stay there the whole day
	Seemed upset/anxious/scared about school
4. Child skipped/wagged/truanted	Headed to school but did not arrive there or left school without permission
5. Parent gave child a day off	To give him/her a rest
6. Parent kept child at home	To help out at home or because school is not helping child
7. Parent arranged extra holidays	Taking a family holiday during school-time
8. Family had an urgent situation	Funeral; someone in the family was taken to hospital
9. Family had other difficulties	Car broke down; someone in the family had a medical appointment
10. Family had religious holiday	Chinese new year or Jewish holidays
11. School was closed	Public holiday/term holidays; curriculum day/teacher training day
12. School sent child home due to his/her behaviour	Was suspended or expelled from school; asked to leave school for the remainder of the day
13. School asked child to stay away	Because the school could not take care of my child’s needs or safe
14. Weather conditions	Snow, floods, fire
15. Other reason	

*Note.* The School Non-Attendance Checklist (SNACK) categorizes school absence types and subtypes based on reasons for a child’s absence. Each category includes examples to clarify the specific nature of absences.

**Figure 2.**
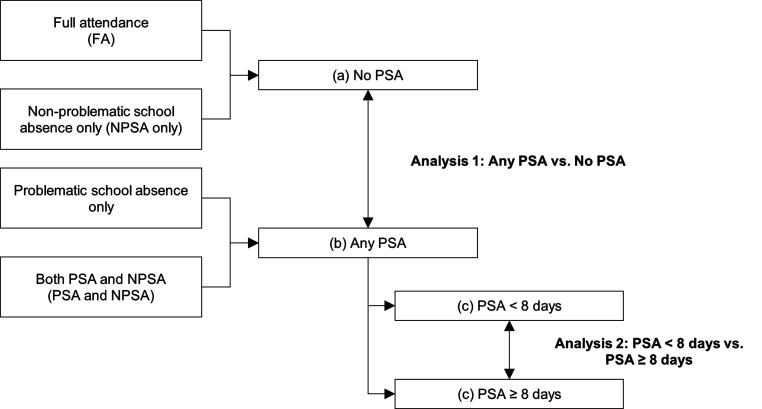
Grouping of Non-problematic School Absence and Problematic School Absence Categories

Based on the NPSA and PSA classifications, patients were grouped into four distinct, mutually exclusive categories: Full attendance (FA), exclusively NPSA (NPSA only), exclusively PSA (PSA only) and both PSA and NPSA (PSA and NPSA). For analyses and in alignment with the approach of Heyne et al. (2019b), the groups were further consolidated into: (a) patients without PSA (comprising “FA” and “NPSA only” cases), (b) patients with any degree of PSA (encompassing “PSA only” and “PSA and NPSA” cases), (c) patients with either no or fewer than 8 days of PSA (PSA <8 days), and (d) patients with eight or more days of PSA (PSA ≥8 days), i.e. ≥ 40% of 20 school days (see also Figure 2). While these groups overlap, it is important to note that groups “a” and “b” are compared in one analysis, whereas groups “c” and “d” are compared in a separate analysis.

The translation of the SNACK tool from English to Dutch and German was executed following a forward-backwards translation protocol adhering to the translation principles outlined by Prieto (1992) to ensure precision and accuracy.

#### Strengths and Mental Health Difficulties

The Strengths and Difficulties Questionnaire parent (SDQ-P) form for children aged 4 – 17 years was used to assess strengths and mental health difficulties (Becker et al., 2018; Goodman, 1997). The questionnaire consisted of 25 questions, each of which was scored on a 3-point Likert scale ranging from 0 as “not true”, 1 as “somewhat true”, and 2 as “certainly true”. The 25 items describe the specific positive and negative attributes of children and adolescents that can be allocated to five scales of five items each: emotional symptoms, conduct problems, hyperactivity-inattention, peer problems, and prosocial behaviours. The SDQ total difficulty score represents the sum of all items, except those from the prosocial behaviours scale. Thus, scores may range from 0 to 40, with a cut-off of more than 16 points deemed “abnormal” (Goodman, 1997).

#### Mental Disorder Classifications

Clinical classifications were determined by a multidisciplinary team of experts on the basis of clinical observations and specific psychological assessments. A child psychiatrist and child psychologist assessed the developmental history, observed the child, conducted a psychiatric assessment, reviewed teacher reports and examined documentation from other professionals involved in the child’s care to reach a consensus diagnosis.

This study used standardized classifications instead of descriptive diagnoses to facilitate consistent comparison. Diagnostic assessments were coded according to the Diagnostic and Statistical Manual of Mental Disorders, Fifth Edition (DSM-5) in the Netherlands (American Psychiatric Association, 2013) and International Classification of Diseases, 10th Revision (ICD-10) criteria in Germany (World Health Organization, 1992). In analogy with San Francisco Health Network (2015) and Dilling and Reinhardt (2015), a DSM-5 to ICD-10 crosswalk was used to convert classifications from the DSM-5 to their corresponding codes in the ICD-10 to compare the prevalence of primary, secondary, and tertiary diagnostic classifications (American Psychiatric Association, 2013). Codes were grouped into broad categories, with automated mapping in SPSS, and manually reviewed by a child and adolescent psychiatrist for accuracy. Classifications were restricted to include the most frequently used subcategories of mental, behavioural and neurodevelopmental disorders with a prevalence of more than 5% in the total sample.

### Participants

Caregivers were invited to participate in a survey after registering their child at child and adolescent psychiatric clinics. A total of 354 caregivers participated from which a total of 354 children and adolescents (159 females, 44.6%), with an average age of 13.0 years (*SD* = 3.5; range 6.2 – 19.9 years), were recruited from Dutch (*n* = 151) and two German (*n* = 203) psychiatric clinics in Viersen and Essen (see Table 2).

**Table 2.** Characteristics of Participants

Characteristics	
Total participants	354
Germany	57.4 (203)
The Netherlands	42.6 (151)
Sex	
Male	55.4 (195)
Female	44.6 (159)
Age	13.0 ± 3.5
Age at school entry	6.0 ± 0.6
School grade	6.4 ± 3.3
SDQ scales	
Total difficulties	19.8 ± 5.6
Emotional symptoms	5.9 ± 2.4
Conduct problems	4.3 ± 2.3
Hyperactivity	5.6 ± 2.4
Peer problems	4.0 ± 2.3
Pro-social behaviour	6.7 ± 2.4
Mental disorder classifications^a^	
Attention-deficit/hyperactivity disorder (F90)	35.6 (126)
Pervasive developmental disorders (F84)	25.1 (89)
Depressive episode (F32)	21.8 (77)
Phobic anxiety disorders (F40)	13.0 (46)
Reaction to severe stress, and adjustment disorders (F43)	10.5 (37)
Conduct disorders (F91)	7.9 (28)
Mixed disorders of conduct and emotions (F92)	7.9 (28)
Cannabis related disorders (F12)	7.6 (27)
Other Behavioural/emotional disorders (F96-F99)	7.6 (27)
Nicotine dependence (F17)	7.1 (25)
Emotional disorders with onset specific to childhood (F93)	7.1 (25)
Comorbidity (average number of comorbid classifications)	2.2 (1.2)

*Note.* Table 2 presents descriptive statistics for the total sample, expressed as % (*n*) or *mean* ± *SD*.

^a^Mental disorder classifications are based on the number of patients with at least one diagnosis from the classifications listed. Only diagnostic classifications with >5% prevalence in the total sample are included.

Children and adolescents were eligible for this study if they were aged 6-20 years, and if they were seeking diagnostic procedures and treatment for mental health problems as outpatients, or were already treated at the clinic as inpatients. The total study sample comprised both inpatients (*n =* 113) and outpatients (*n =* 241). The majority of responding caregivers were parents: 276 mothers (78.0%), 40 fathers (11.3%), while those remaining were foster parents or other caregivers. Parents completed Strengths and Difficulties Questionnaire (SDQ) scores showed high levels of total difficulties, emotional symptoms, conduct problems, peer problems and prosocial behaviour, and close to average hyperactivity scores. Classifications of attention-deficit/hyperactivity disorder were most common, followed by pervasive developmental disorders, depressive episodes and phobic anxiety disorders. Patients had an average of 2.2 classifications.

### Statistical Methods

All analyses were conducted using SPSS version 25. Associations between sample characteristics and PSA were assessed using a stepwise logistic regression model. Stepwise logistic regression analysis elucidated the association between PSA and the following independent variables: In step 1, age, sex, recruitment site (with Nijmegen as a reference), and ISCED level and treatment modality (inpatient vs. outpatient) were chosen as control variables. Additionally, the SDQ (step 2), classifications (step 3) and the number of classification (step 4) were entered into the model. Two models were estimated: Model A estimates the odds ratio for “Any PSA” vs. “No PSA”. Model B compares patients with no absence or problematic school absence for less than eight days to those with problematic school absence for more than or equal to eight days (75th percentile; “PSA <8 days” vs. “PSA ≥8 days”). The 75th percentile benchmark indicates an absence of 40% (out of 20 days). This benchmark is well above the 10% suggested by Kearney and Graczyk (2020) to demarcate chronic school absence, and was used to facilitate comparative analyses aimed at identifying longer duration of PSA (Ialongo, 2019).

## Results

### Overall Levels of School Absence

**Figure 3.**
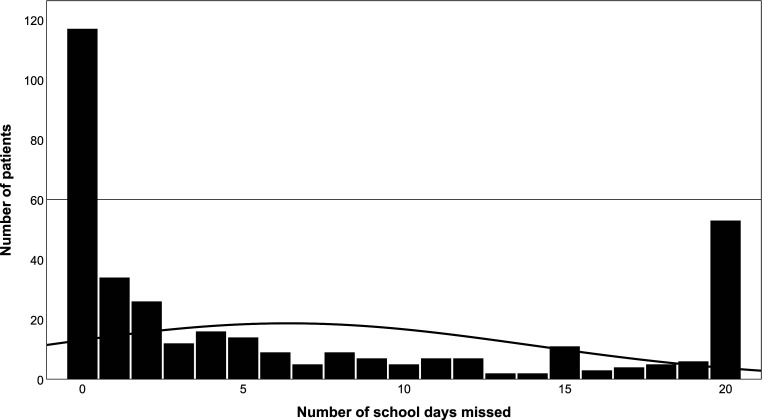
Distribution of Number of School Days Missed by Participants

Figure 3 shows the average numbers of school days missed during the data collection period. Out of the 20 school days, participants missed on average 6 days (*M* = 6.40; *SD* = 7.58; range: 0–20). The median of school days missed was 2.25. Overall, 66.9% (*n* = 237) of the participants missed 1 day or more of school. Fifteen percent (*n* = 53) of patients did not attend school at all during the 20 school days. Chronic school absence was reported in 50.0% (*n* = 176) of children and adolescents in this study, including both NPSA and PSA cases. Moreover, using the 75^th^ percentile benchmark (i.e. 40% problematic school absence) showed 34.2% (*n* = 121) of the participants with PSA did not attend school for 8 days or more.

### Non-Problematic and Problematic School Absence

Table 3 presents the distribution of the number of school days missed during the 20 school days prior to inclusion. Among the participants, 33.1% (*n =* 117) did not miss any school days. A total of 24.8% (*n =* 88) of children were absent between 1 and 5 days, while 12.4% (*n =* 44) missed between 5 and 10 days of school. Absences between 10 and 15 days were noted in 6.5% (*n =* 23) of the patients. Notably, 23.2% (*n =* 82) of youths were reported absent between 15 and 20 out of the past 20 school days. NPSA was identified in 42.9% (*n =* 152) of participants. Similarly, 42.1% (*n =* 149) of patients missed school due to PSA. School refusal and school exclusion were among the most prevalent types of PSA followed by truancy and school withdrawal. Furthermore, 25.4% (*n* = 90) of patients missed 8 days of school due to PSA, and 9.6% (*n* = 34) did not attend school at all because of PSA.

**Table 3.** Non-Problematic and Problematic School Absence

	Total (*N* = 354)
Days absent from school in the past 20 school days	
0	33.1 (117)
1-5	24.8 (88)
5-10	12.4 (44)
10-15	6.5 (23)
15-20	23.2 (82)
NPSA	42.9 (152)
PSA^+^	42.1 (149)
School refusal	21.8 (77)
School truancy	7.3 (26)
School withdrawal	6.5 (23)
School exclusion	20.9 (74)
Chronic school absence^‡^	50.0 (176)
Severe PSA	
≥8 days of PSA (75^th^ percentile)	25.4 (90)
20 days of PSA only	9.6 (34)

*Note.* NPSA = non-problematic school absence; PSA = problematic school absence. Values are presented as frequency and number of patients % (*n*). ^+^ Parents reported a minimum of a half day for inclusion in this category of these PA-subtypes. ^‡^ Chronic school absence is defined as any absence of more than 10% of school days.

### Associations Between Problematic School Absence and Sample Characteristics

A stepwise regression analysis was used to differentiate between the effects of mental health strengths and difficulties as measured by SDQ, and classification. Two models were used to examine the relationships between PSA incidence and severity and between PSA incidence and the independent variables.

#### Model A: “Any PSA” vs. “No PSA”

In Model A1 (Table 4) testing for any PSA versus no PSA, a slightly higher odds ratio was found for older patients, indicating that age positively predicts having any PSA. Inpatient treatment showed a significantly lower odds ratio for no PSA, implying inpatients have a higher risk of any PSA.

**Table 4.** Associations Between Prevalence of Problematic School Absence (PSA) and Sociodemographic Variables, SDQ and Diagnostic Classifications Testing for “Any PSA” vs. “No PSA”

Covariates	Model A1				Model A2				Model A3				Model A4			
	*B*	*OR*	*95% CI*	*p*	*B*	*OR*	*95% CI*	*p*	*B*	*OR*	*95% CI*	*p*	*B*	*OR*	*95% CI*	*P*
**Age**	**0.21**	**1.23**	**1.07** **- 1.42**	**0.003**	**0.23**	**1.26**	**1.09** **- 1.46**	**0.002**	**0.17**	**1.18**	**1.00** **- 1.39**	**0.045**	0.08	1.09	0.92 **-** 1.29	0.346
Sex	0.07	1.08	0.63 - 1.84	0.786	0.09	1.09	0.62 **-** 1.93	0.761	0.40	1.49	0.79 **-** 2.83	0.219	0.33	1.40	0.73 **-** 2.67	0.314
Viersen	−0.19	0.83	0.36 - 1.89	0.651	−0.16	0.86	0.36 **-** 2.04	0.727	−0.12	0.89	0.35 **-** 2.29	0.805	−0.13	0.88	0.33 **-** 2.32	0.793
Essen	0.01	1.01	0.48 - 2.14	0.977	0.16	1.17	0.52 **-** 2.64	0.702	0.23	1.26	0.51 **-** 3.13	0.615	0.41	1.51	0.60 **-** 3.77	0.378
ISCED-level 2	−0.25	0.78	0.30 - 2.05	0.616	−0.19	0.83	0.30 **-** 2.25	0.708	−0.15	0.86	0.30 **-** 2.52	0.785	−0.55	0.58	0.19 **-** 1.73	0.328
ISCED-level 3	−0.48	0.62	0.17 - 2.28	0.472	−0.38	0.68	0.18 **-** 2.65	0.580	−0.43	0.65	0.15 **-** 2.82	0.565	−0.84	0.43	0.10 **-** 1.93	0.272
**Treatment modality (Inpatient)** ^a^	**−1.68**	**0.19**	**0.09** **- 0.38**	**0.000**	**−1.75**	**0.17**	**0.08** **- 0.36**	**0.000**	**−1.87**	**0.15**	**0.07** **- 0.34**	**0.000**	**−1.76**	**0.17**	**0.08** **- 0.39**	**0.000**
SDQ - Emotional symptoms					0.10	1.11	0.98 **-** 1.25	0.090	0.05	1.05	0.92 **-** 1.19	0.467	0.04	1.04	0.92 **-** 1.19	0.509
**SDQ - Conduct problems**					**0.15**	**1.16**	**1.01** **- 1.33**	**0.032**	**0.22**	**1.24**	**1.07** **- 1.44**	**0.005**	**0.19**	**1.21**	**1.04** **- 1.41**	**0.014**
SDQ - Hyperactivity/Inattention					0.04	1.04	0.92 **-** 1.18	0.519	0.08	1.09	0.95 **-** 1.25	0.236	0.08	1.09	0.94 **-** 1.25	0.245
SDQ - Peer problems					0.06	1.07	0.94 **-** 1.21	0.317	0.01	1.01	0.88 **-** 1.15	0.936	0.02	1.02	0.89 **-** 1.17	0.753
SDQ - Prosocial behaviour					0.02	1.02	0.90 **-** 1.16	0.726	0.05	1.05	0.92 **-** 1.20	0.486	0.03	1.03	0.90 **-** 1.18	0.685
**Attention-deficit hyperactivity disorders (F90)**									−0.39	0.68	0.33 - 1.41	0.301	**−0.83**	**0.44**	**0.19** **- 0.99**	**0.046**
Pervasive development disorders (F84)									0.46	1.59	0.71 **-** 3.58	0.264	0.10	1.11	0.48 **-** 2.59	0.809
**Depressive episode (F32)**									**0.98**	**2.66**	**1.21** **- 5.85**	**0.015**	0.66	1.93	0.84 **-** 4.42	0.121
**Phobic anxiety disorders (F40)**									**1.20**	**3.33**	**1.30** **- 8.52**	**0.012**	0.67	1.95	0.72 **-** 5.27	0.187
Reaction to severe stress, and adjustment disorders (F43)									0.57	1.77	0.71 **-** 4.43	0.225	0.34	1.41	0.54 **-** 3.66	0.486
Conduct disorders (F91)									−0.13	0.88	0.31 **-** 2.49	0.812	−0.58	0.56	0.18 **-** 1.71	0.309
Mixed disorders of conduct and emotions (F92)									−0.08	0.92	0.31 **-** 2.71	0.880	−0.32	0.72	0.24 **-** 2.21	0.569
**Other behavioural and emotional disorders (F96-99)**									**−1.25**	**0.29**	**0.09** **- 0.90**	**0.033**	**−1.82**	**0.16**	**0.05** **- 0.57**	**0.005**
Emotional disorders with onset specific to childhood (F93)									0.01	1.01	0.34 **-** 3.02	0.98	−0.34	0.71	0.23 **-** 2.16	0.546
**Comorbidity**													**0.58**	**1.78**	**1.25** **- 2.54**	**0.002**
*Nagelkerke's R2*	0.35				0.39				0.45				0.48			

*Note.* A stepwise logistic regression analysis was used to test for associations between problematic school absence and potential confounders, the SDQ-scales and broad ICD-10 classifications. Nijmegen was used as a reference for testing between recruitment centres. Significance levels at *p* < 0.05, *p* < 0.01 and *p* < 0.001 were found, with significant covariates highlighted in bold. OR = Odds Ratio; CI = Confidence Interval.

^a^Negative B values indicate higher odds of problematic school absence for inpatients.

The inclusion of SDQ variables in model A2 revealed a higher risk for subjects scoring high on the conduct problems scale, suggesting that elevated conduct problems increase the likelihood of PSA.

Model A3, which incorporates the most prevalent diagnostic classifications, showed that depressive episodes and phobic anxiety disorders were significantly associated with any PSA. Patients with these classifications had an approximately threefold higher odds of not attending school due to PSA reasons. Additionally, higher age, inpatient treatment, and high SDQ scores on the conduct problems scale remained significantly associated with a greater likelihood of PSA.

The introduction of the comorbidity per subject in the final Model A4 revealed a high odds ratio, indicating that each additional classification increases the risk of any PSA by approximately twofold. While higher age, depressive episodes, and phobic anxiety were no longer significantly associated with PSA, inpatient treatment, and SDQ conduct problems remained significant predictors for PSA. Conversely, subjects with the classifications “other behavioural and emotional disorders” and those with attention-deficit/hyperactivity disorder had a lower risk of any PSA.

#### Model B: “PSA <8 Days” vs. “PSA ≥8 Days”

Analysing the severity of PSA, Model B (Table 5) presents the logistic regression model estimating odds ratios for the same set of independent variables as used in Model A. Similar to Model A, higher age and inpatient treatment significantly predicted PSA of eight or more days.

**Table 5.** Associations Between Severity of Problematic School Absence (PSA) and Sociodemographic Variables, SDQ and Diagnostic Classification Testing for “PSA <8 Days” vs. “PSA ≥8 Days”

Covariates	Model B1				Model B2				Model B3				Model B4			
	*B*	*OR*	*95% CI*	*p*	*B*	*OR*	*95% CI*	*p*	*B*	*OR*	*95% CI*	*p*	*B*	*OR*	*95% CI*	*p*
**Age**	**0.35**	**1.42**	**1.21** **- 1.68**	**0.000**	**0.38**	**1.46**	**1.23** **- 1.74**	**0.000**	**0.34**	**1.40**	**1.15** **- 1.72**	**0.001**	**0.32**	**1.38**	**1.12** **- 1.69**	**0.002**
Sex	0.26	1.30	0.71 - 2.35	0.395	0.33	1.40	0.74 - 2.64	0.305	0.65	1.92	0.93 - 3.96	0.078	0.62	1.85	0.90 - 3.83	0.096
Viersen	−0.61	0.54	0.18 - 1.61	0.272	−0.62	0.54	0.18 - 1.62	0.270	−0.97	0.38	0.11 - 1.34	0.131	−0.96	0.38	0.11 - 1.37	0.140
Essen	−0.54	0.59	0.21 - 1.61	0.300	−0.55	0.58	0.20 - 1.66	0.308	−0.42	0.65	0.21 - 2.09	0.474	−0.35	0.71	0.22 - 2.28	0.562
ISCED-level 2	0.39	1.48	0.45 - 4.91	0.520	0.51	1.67	0.49 - 5.64	0.410	0.79	2.20	0.58 - 8.31	0.246	0.66	1.94	0.51 - 7.38	0.332
ISCED-level 3	1.09	2.97	0.59 - 15.01	0.187	1.34	3.82	0.73 -20.06	0.113	1.55	4.73	0.81 - 27.66	0.084	1.41	4.11	0.70 - 24.23	0.118
**Treatment modality (inpatient)^a^**	**−1.93**	**0.15**	**0.06** **- 0.37**	**0.000**	**−1.96**	**0.14**	**0.06** **- 0.36**	**0.000**	**−1.85**	**0.16**	**0.06** **- 0.44**	**0.000**	**−1.87**	**0.16**	**0.06** **- 0.43**	**0.000**
**SDQ - Emotional symptoms**					**0.14**	**1.15**	**1.00** **- 1.32**	**0.043**	0.07	1.08	0.93 - 1.25	0.339	0.07	1.07	0.92 - 1.25	0.393
SDQ - Conduct problems					0.06	1.06	0.92 - 1.22	0.447	0.09	1.09	0.93 - 1.29	0.287	0.08	1.09	0.92 - 1.28	0.333
SDQ - Hyperactivity/Inattention					0.02	1.02	0.89 - 1.17	0.802	0.05	1.05	0.90 - 1.22	0.546	0.05	1.05	0.90 - 1.22	0.528
SDQ - Peer problems					0.01	1.01	0.88 - 1.16	0.908	−0.04	0.96	0.82 - 1.13	0.645	−0.03	0.97	0.83 - 1.13	0.673
SDQ - Prosocial behaviour					−0.01	0.99	0.87 - 1.14	0.906	0.01	1.01	0.87 - 1.17	0.935	0.01	1.01	0.87 - 1.17	0.924
Attention-deficit hyperactivity disorders (F90)									−0.43	0.65	0.27 - 1.58	0.343	−0.58	0.56	0.22 - 1.41	0.219
Pervasive development disorders (F84)									0.41	1.50	0.57 - 3.94	0.412	0.26	1.30	0.48 - 3.54	0.605
**Depressive episode (F32)**									**0.84**	**2.31**	**1.04** **- 5.12**	**0.040**	0.63	1.88	0.79 - 4.50	0.154
**Phobic anxiety disorders (F40)**									**1.19**	**3.28**	**1.39** **- 7.75**	**0.007**	**0.95**	**2.59**	**1.00** **- 6.70**	**0.050**
Reaction to severe stress, and adjustment disorders (F43)									0.26	1.30	0.45 - 3.77	0.628	0.13	1.14	0.38 - 3.42	0.814
Conduct disorders (F91)									−0.35	0.71	0.20 - 2.46	0.586	−0.55	0.58	0.16 - 2.14	0.412
Mixed disorders of conduct and emotions (F92)									1.01	2.75	0.84 - 9.02	0.095	0.89	2.44	0.73 - 8.16	0.147
Disorder due to use of cannabis (F12)									−0.88	0.41	0.06 - 3.10	0.391	−1.01	0.36	0.05 - 2.86	0.336
Other behavioural and emotional disorders (F96-99)									−0.34	0.71	0.20 - 2.54	0.603	−0.50	0.60	0.17 - 2.21	0.447
Disorder due to use of nicotine (F17)									1.65	5.20	0.67 - 40.43	0.115	1.27	3.55	0.40 - 31.45	0.255
Emotional disorders with onset specific to childhood (F93)									−0.36	0.70	0.19 - 2.56	0.590	−0.52	0.60	0.16 - 2.23	0.440
Comorbidity													0.23	1.26	0.85 - 1.86	0.260
*Nagelkerke's R* ^*2*^	0.33				0.35				0.42				0.42			

*Note.* A stepwise logistic regression analysis was used to test for associations between problematic school absence and potential confounders, the SDQ-scales and broad ICD-10 classifications. Nijmegen was used as a reference for testing between recruitment centres. Significance levels at *p* < 0.05, *p* < 0.01 and *p* < 0.001 were found, with significant covariates highlighted in bold. OR = Odds Ratio; CI = Confidence.

^a^Negative B values indicate higher odds of severe problematic school absence for inpatients.

The inclusion of SDQ variables in model B2 revealed a statistically significant association with “emotional symptoms”, suggesting that higher emotional symptoms scores are linked to an increased risk of severe PSA. The association of higher age and inpatient treatment with severe PSA remained.

In model B3, which incorporates the most prevalent diagnostic classifications, depressive episodes and phobic anxiety disorders significantly predicted severe PSA. Higher age and inpatient treatment remained significant, whereas SDQ emotional symptoms were no longer statistically significant.

In the full B4 model which includes comorbidity, higher age, inpatient treatment and phobic anxiety disorders were associated with more severe PSA. However, the inclusion of the comorbidity rendered the previously significant predictor of depressive episode and severe PSA non-significant.

## Discussion

This study explored problematic school absence among Dutch and German children and adolescents with mental health problems. The study assessed associations with high prevalence and duration of problematic school absence in a clinical sample. Data indicate that problematic school absence were highly prevalent among these children and adolescents. Two-thirds of the total sample reported problematic school absence, with half having at least three days of problematic school absence, and almost a quarter exceeding eight days in the four weeks before inclusion. Furthermore, inpatient treatment, conduct problems, and comorbidity were significantly correlated with showing any problematic school absence. In addition, the risk of severe problematic school absence were predicted by higher age, inpatient treatment and phobic anxiety disorders.

On average, children and adolescents in this study missed 6 out of 20 school days. Over two-thirds of the students missed at least one day. This absent rate is considerably higher compared to general populations in the Netherlands and Germany (9.6% and 8.7%, respectively), indicating that this is a serious problem for young people with mental health problems (Dutch Central Bureau for Statistics, 2018; Pflug & Schneider, 2016). Given that our data used a clinical population sample with more mental health problems, this finding is in line with expectations.

Although marginally less prevalent than non-problematic school absence, this study found high problematic school absence rates, with 42.1% citing a problematic school absence reason. Reasons for school absence varied. School refusal and school exclusion were the most prevalent reasons, with 21.8% and 20.8% respectively. Less frequent were truancy with 7.3% and school withdrawal with 6.5%. While school refusal has been reported to be associated with psychiatric disorders in other studies (Egger et al., 2003), our findings showed significantly higher rates of school refusal (21.8% vs. 2.0%). This is most likely because their population had relatively mild school refusal and three quarters of students did not meet criteria for any psychiatric disorders (Egger et al., 2003). Similar to school refusal, school exclusion is significantly associated with psychopathology, and psychopathology with exclusion (Ford et al., 2018). In particular, children with conduct disorders (Parker et al., 2015) and depressive episodes (Rushton et al., 2002) seem to be at higher risk for school exclusion. Given the severity of mental health problems in the population in this study compared to general populations, higher rates of problematic school absence types are expected.

This research showed that higher age was associated with a higher likelihood of severe problematic school absence. This finding is supported by existing literature, extending to non-psychiatric samples, indicating that older youths tend to have lower school attendance rates (Henry, 2007; Hunt & Hopko, 2009). The higher prevalence of problematic school absence in these adolescents may be attributed to their increasing autonomy and diminished parental control during this developmental phase (Steinberg, 1987).

Inpatient care was significantly associated with both any problematic school absence and severe problematic school absence. Specifically, the odds of having any problematic school absence were 83% higher for the inpatients compared to outpatients. Likewise, the odds of severe problematic school absence were 84% higher for inpatients than for outpatients. This finding may be explained by the participants’ inability to attend school because of more severe behavioural problems and multiple classifications (McDermott et al., 2002). However, further research is needed to elucidate these effects.

Results of this study showed that SDQ conduct problems increase the risk of severe problematic school absence. Previous studies have shown that conduct problems are known to be linked to school refusal, truancy and school exclusion (Egger et al., 2003; Kearney, 2008b; Parker et al., 2015). A possible explanation might be that school are more likely to set boundaries in response to children’s disruptive behaviour from conduct problems. This, in turn, may increase the likelihood of school exclusion and refusal.

Problematic school absence was more prevalent among patients with comorbidities, with each additional classification nearly doubling the risk of any problematic school absence. This finding aligns with previous research, which indicates that students with multiple psychiatric disorders are at a significantly higher risk of problematic school absence compared to those with a single classification (John et al., 2022). A possible reason is that having more than one condition leads to a wider range of symptoms and difficulties and therefore, a more severe psychiatric condition (Zimmerman et al., 2018). These challenges can compound, leading to more severe symptoms and making school attendance more challenging.

This study found significant associations between having an anxiety disorder and severe problematic school absence. Previous studies have shown anxiety related disorders to be associated with greater odds of having problematic school absence (Finning et al., 2019; Kearney & Albano, 2004). In a systematic review, Finning et al. (2019) have suggested anxiety is linked to particular forms of school absence, such as truancy and school refusal. As such, our findings are in line with the existing literature.

This study found that attention-deficit/hyperactivity disorder was negatively associated with problematic school absence, suggesting that it may be linked to a lower risks of absence. This finding contrasts with previous research indicating that attention-deficit/hyperactivity disorder is typically associated with poorer school attendance (May et al., 2021; Niemi et al., 2022). One possible explanation is that many of the participants with attention-deficit/hyperactivity disorder in this study receive medication as part of their treatment, which may help mitigate its negative impact on school attendance. Research has shown medication reduces school absence and improve academic performance (Barbaresi et al., 2007). However, the long-term effects of medication on school attendance remain less conclusive (Langberg & Becker, 2012).

Although this study was conducted in 2019, before the COVID-19 pandemic, the findings remain relevant in the post-pandemic context. Research shows that school attendance dropped markedly during and after the pandemic, with chronic absence rates almost doubling compared to pre-pandemic years and academic outcomes declining (Swiderski et al., 2024, 2025). In addition, the re-entry to school after long periods of virtual learning or school closures has heightened anxiety, depression, and stress for many young people (Krause et al., 2025; Milaniak et al., 2024). Meta-analytic evidence shows that clinically elevated anxiety symptoms nearly doubled during the pandemic, and even adolescents with little or no prior history of anxiety suffered from significant distress (Hawes et al., 2021; Madigan et al., 2023). This study identified important mental health vulnerabilities that overlap with those of the children and adolescents now affected most in the post-pandemic period. The results of this study provide valuable insight into which children were already at risk before the pandemic. These same groups are now especially vulnerable, as post-pandemic stressors such as re-entry anxiety and worsened mental health place them at even higher risk for persistent school absence. This underscores the importance of addressing problematic school absence in vulnerable groups.

These findings point to several practical steps for clinicians and schools. Routine screening for school attendance problems should be integrated into psychiatric assessment, particularly for high-risk groups such as inpatient adolescents with higher age, conduct problems, phobic anxiety disorders, and multiple classifications. Treatment plans should not only address symptom reduction but also include clear goals for school reintegration, for example through gradual exposure or family-based support. Close collaboration between mental health services and schools services is essential. It enables the possibility of early referral to child and adolescent psychiatry for diagnostic procedures and reduce prolonged absence. It also supports re-entry after extended school disruption and helps prevent the vicious cycle of school absence leading to mental health problems and vice versa, reinforcing each other. Finally, further international research is needed to better understand and address problematic school absence in child and adolescent psychiatry.

This study included a large number of patients from three recruitment sites from two countries, however, it has several limitations. First, up to date the SNACK has been used in only a few clinical samples. The theoretically grounded scales of the SNACK make classical test validation difficult. Although a translation back-translation protocol using a certified translator has been rigorously followed, the validity of Dutch and German versions has not yet been investigated. Nevertheless, preliminary results from unpublished data indicate a strong association between SNACK subtypes and the expected ICD-10 classification, suggesting that the translated versions yield consistent outcomes. Nonetheless, to ensure full validation in Dutch and German contexts, more rigorous validation studies are necessary. Second, this study compared convenience samples, which may have influenced the representativeness of our findings. Third, this study used a convenience sampling selecting participants who were readily available, which may have led to a sampling bias. Assessing representativeness, in the Nijmegen sample, the average age was slightly higher (*T* = 2.66; *p* = 0.008), and the prevalence of schizophrenia patients was lower than expected (*χ*^*2*^ = 40.97; *p* = 0.000). The Viersen outpatient sample had fewer males than expected (*χ*^*2*^ = 3.85; *p* = 0.05), while the inpatient study sample showed lower rates of depressive and anxiety disorders and higher rates of problematic substance use (*χ*^*2*^ = 56.79; *p* = 0.000). The Essen study sample showed no differences from the overall Essen patient population (outpatient and inpatient). Fourth, for practical reasons, this study relied on parent-reported school attendance, rather than official school records. The parents’ potential misinformation regarding their children’s school attendance may have biased the results. Finally, apart from school attendance, the study did not include other social factors such as government policies, economic status, quality of healthcare, or family structure and dynamics. While this study provides valuable insights into problematic school absence child and adolescent psychiatry, its outcomes are explorative and descriptive. Future research should focus on identifying social factors that may influence these problems, and investigate protective and risk factors for problematic school absence in child and adolescent psychiatry.

## Conclusions

In sum, we explored problematic school absence and potential risk factors that may predict problematic school absence for children and adolescents receiving mental health care, in an effort to create awareness among clinicians and provide a foundation for future research. To the best of our knowledge, this is the first study to investigate problematic school absence in child and adolescent psychiatry across two adjacent countries. The findings of this study show that problematic school absence is highly prevalent in these children and adolescents. Furthermore, this study using an international sample provides insights into the factors that may be associated with problematic school absence in children and adolescents with mental health problems. Inpatient treatment modality, SDQ conduct problems and comorbidity were predictive for any problematic school absence. Furthermore, higher age, inpatient treatment modality, and phobic anxiety disorders predicted severe problematic school absence. As such, this study sheds light on the severity of problematic school absence in clinical psychiatric populations.

## Data Availability

Anonymous data and SPSS syntax codes are available from the corresponding author upon reasonable request.*

## Abbreviations

LVR: Landschaftsverband Rheinland
SNACK: School-Non-Attendance ChecKlist
PSA: Problematic school absence
NPSA: Non-problematic school absence
FA: Full attendance
SDQ: Strengths and Difficulties Questionnaire
DSM-5: Diagnostic and Statistical Manual of Mental Disorders, Fifth Edition
ICD-10: International Classification of Diseases, 10th Revision
SPSS: Statistical Package for the Social Sciences
ANOVA: Analysis of variance
ISCED: International Standard Classification of Education
OR: Odds ratio
CI: Confidence Interval
M: Mean
SD: Standard deviation
